# School-Based Diabetes Care: A National Survey of U.S. Pediatric Diabetes Providers

**DOI:** 10.1155/2023/4313875

**Published:** 2023-04-11

**Authors:** Christine A. March, Linda M. Siminerio, Traci M. Kazmerski, Anastasia Albanese-O'Neill, Elizabeth Miller, Ingrid Libman

**Affiliations:** ^1^Division of Pediatric Endocrinology, UPMC Children's Hospital of Pittsburgh, University of Pittsburgh, Pittsburgh, PA, USA; ^2^Department of Medicine, University of Pittsburgh, Pittsburgh, PA, USA; ^3^Division of Adolescent and Young Adult Medicine, UPMC Children's Hospital of Pittsburgh, University of Pittsburgh, Pittsburgh, PA, USA; ^4^Department of Family, Community and Health System Science, University of Florida College of Nursing, Gainesville, Florida, USA

## Abstract

**Objectives:**

To understand the practices, attitudes, and beliefs of type 1 diabetes (T1D) providers towards school-based diabetes care (SBDC), including counseling families and communicating with schools, and explore the barriers and facilitators which affect their support of SBDC. *Research Design and Methods*. We conducted a national survey of pediatric T1D providers about their perceived support of SBDC, including family counseling and school communication. We used descriptive statistics to analyze results and explored differences by practice size (<500, 500–999, and ≥1000 patients) and environment (academic vs nonacademic).

**Results:**

A total of 149 providers completed the survey. Nearly, all (95%) indicated SBDC was very important. Though most (63%) reported counseling families about SBDC multiple times per year, few (19%) spoke with school staff routinely, reporting that was a shared responsibility among different providers. Close to 90% agreed school feedback on T1D management plans would be helpful, yet only 31% routinely requested this input. Moderate to extremely significant barriers to SBDC communication included internal factors, such as staff resources (67%) and time (82%), and external factors, such as school nurse education needs (62%) and differing school district policies (70%). Individuals from large or academic practices reported more barriers in their knowledge of SBDC, including federal/state laws. Desired facilitators for SBDC included a designated school liaison (84%), electronic transmission for school forms (90%), and accessible school staff education (95%).

**Conclusions:**

Though providers universally agree that SBDC is important, there are multilevel internal (practice) and external (policy) barriers to facilitating a bidirectional relationship between schools and health teams.

## 1. Introduction

Over the last few decades, there have been numerous advances in the management of type 1 diabetes (T1D), with the development of new insulin analogs and increasing use of technologies, including insulin pumps, continuous glucose monitors (CGMs), and automated insulin delivery systems. Though these advances may improve glycemic control [1, 2], they also increase the complexity of treatment plans. For children with T1D, this affects not only their caregivers at home but also their support staff in school.

In the United States, school nurses or other trained personnel often supervise T1D management in school, helping to provide these children with a safe environment and equal opportunities for learning [3]. In their role, school nurses should help foster communication between the child's family and medical team to deliver consistent, quality care [4]. For children with chronic health conditions, school nurse-led care coordination has been shown to reduce gaps in access and improve related health outcomes [5]. School-based asthma programs are perhaps the best studied. Interventions focusing on developing partnerships and/or telemedicine to establish communication between the family, school nurse, and clinicians have been found to be the most impactful approaches for improving medication adherence and reducing asthma-related symptoms and acute care utilization [6–8]. Studies are more limited in pediatric T1D, though pilot interventions in school nurse case management or school-based telemedicine have been found to improve diabetes-related quality of life [9–11].

There have been many scientific inquiries into how effectively health-care providers communicate with school health staff on behalf of children with T1D [12–17]. Findings from these studies show that though the exchange of information is well-received [18], school nurses and parents often perceive that this communication is insufficient. There is a dearth of literature on T1D health-care provider perspectives on interactions with schools. To foster best practices for school-provider collaborative interventions and evaluate the impact on diabetes-related health outcomes for children, a better understanding of providers' perceived support of school-based diabetes care (SBDC) is needed.

The primary goal of this study was to understand the current practices, attitudes, and beliefs of pediatric T1D providers towards components of SBDC, including counseling patients and families and communicating with school nurses and other health staff. A secondary goal was to explore the perceived impact of various factors which may impede providers' ability to participate in SBDC (e.g., barriers) and how helpful different strategies may be to overcoming those barriers (e.g., facilitators). This information may guide strategies to optimize school-provider collaboration from a health systems level.

## 2. Methods

We conducted a cross-sectional online survey of pediatric T1D providers, including physicians and advanced practice providers (APP, includes nurse practitioners and physician assistants), in the United States who currently participate in the care of children with T1D. The survey evaluated current practices supporting SBDC and their perceptions (attitudes and beliefs) for their role in SBDC. We defined SBDC as any aspect of diabetes management for a child that may occur in the school setting. Additional questions were asked about the barriers and facilitators to their participation in SBDC. Barriers referred to the internal (individual, practice) or external (school, policy) factors which negatively influence their current participation in SBDC. Facilitators referred to potential strategies to overcome the included barriers.

### 2.1. Context

In the United States, federal laws, including the Americans with Disabilities Act, Section 504 of the Rehabilitation Act, and Individuals with Disabilities Education Act [19–22], protect the rights of children with diabetes in schools which receive federal funding. These laws require individualized assessments, reasonable accommodations, and the establishment of written diabetes care plans, enabling children to fully participate in all school activities. Many states have additional regulations which specifically address aspects of diabetes management (e.g., blood glucose monitoring and medication administration) in school for both school nurses and nonmedical school staff [3]. In many, but not all, states, nonmedical staff are permitted to administer insulin and glucagon under state law; however, most school systems rely on school nurses to provide direct medical care, conduct needed health screenings, develop individualized care plans, and advocate for students' health needs [23]. As such, school nurses are often the primary point of contact for parents and health-care providers in the school setting.

### 2.2. Survey Development

The investigator-developed survey consisted of 30 questions which were grouped into categories addressing different types of support for SBDC: counseling families, communicating with school staff, training school staff, barriers, and facilitators to their participation in SBDC, and participant and practice background characteristics. Questions followed a multiple choice or Likert scale format, with some open-ended responses available to justify a selection of “other” or provide clarifying comments (optional). The survey is available in the online supplement.

Prior to distribution, we piloted the survey using cognitive interviewing with five experts in SBDC (two physicians, two APPs, and one diabetes care and education specialist (DCES)) to establish content validity. We asked providers to “think aloud” as they read and answer each question to identify problems with word choice, clarity, or relevance to the research goal [24]. We used verbal probes to understand question comprehension. We took detailed notes which were reviewed by the research team and an existing group of community partners, consisting of providers, school nurses, and parents of children with T1D [25]. We revised questions following each interview until no new concerns arose prior to distribution.

### 2.3. Distribution

We distributed the survey by anonymous link through the newsletters or email list serv for three organizations to reach our target audience: the American Diabetes Association (ADA) Diabetes in Youth Interest Group, the T1D Exchange Quality Improvement Collaborative, and the Pediatric Endocrine Society. One organization requested slight modifications to the questionnaire, including the addition of one question and word changes to two questions which did not affect the nature of the question asked. These changes are noted in the results where appropriate.

We collected survey responses between November 2021 and April 2022. We incentivized participation with the chance to win one of ten $200 gift cards. Prior to taking the survey, we informed participants of the objective, time burden, and potential risks and asked them to indicate consent. We prevented respondents from re-accessing the link by the same email address to avert duplicate responses. All surveys were completed in Qualtrics (Provo, Utah). The University of Pittsburgh Institutional Review Board deemed this study exempt (PRO#20100457).

### 2.4. Analysis

We calculated descriptive statistics for all items. We summarized Likert scale responses by mean score and standard deviation and/or the number and percentage answering in agreement (response options 4 or 5). Moderately to extremely significant barriers or facilitators (response options 3–5) are reported as a number and percentage. We used Chi-square or Fisher's exact test for categorical data and the Mann–Whitney *U* test or Kruskal–Wallis test for ordinal data to analyze differences in responses by practice size (small: less than 500 patients, medium: 500–999 patients, and large: 1000 patients or more) and practice environment (academic vs nonacademic). All analyses were completed in Stata version 17 (StataCorp LLC, College Station, Texas) with significance determined by a *p* value of <0.05. Small amounts of missing data are reported in the tables (range 0–3 for survey responses, 0–16 for background questions). As few respondents answered optional open-ended questions, these data were not included as it did not add to the findings.

## 3. Results

### 3.1. Summary of Respondents

Of 180 surveys received, 31 were excluded due to missing >50% of responses, leaving 149 for analyses. Among those excluded, most (*n* = 18, 58%) were opened, and no questions were completed; the remainder (*n* = 13, 42%) were partially completed. Most respondents were physicians (*n* = 133, 89%). Mean age was 44.7 ± 11.9 years (range 25–80). The majority identified as female (*n* = 110, 74%), 25% as male (*n* = 38), and 1% as nonbinary (*n* = 1). Self-reported race and ethnicity included 68% non-Hispanic White (*n* = 101), 17% Asian (*n* = 25), 7% Hispanic or Latin-x (*n* = 11), 2% Black or African American (*n* = 3), 3% biracial or multiracial (*n* = 5), and 3% who declined to answer (*n* = 4).

Respondents' practice settings varied, including location, environment, size, types of staff employed, and their years of experience in their role (Table 1). Most (*n* = 123, 83%) reported having the combined support of a clinician, DCES, and dietician, and nearly as many had behavioral health support (*n* = 121, 81%). Compared with mid- or large-sized practices, individuals working in small practices were slightly less likely to have a DCES (small 74%, medium 93%, large 93%, *p*=0.01), dietician (small 71%, medium 95%, large 97%, *p* < 0.001), psychologist (small 23%, medium 55%, large 71%, *p* < 0.001), or social worker (small 34%, medium 70%, large 90%, *p* < 0.001) on staff. Half of respondents working in small practices reported an academic practice environment (*n* = 19, 54%), compared with 83% of medium (*n* = 34) and 89% of large (*n* = 64) practices (*p* < 0.001).

### 3.2. Counseling Families about SBDC

There was near unanimous agreement (*n* = 141, 95%) among providers that SBDC is very or extremely important for their patients. Counseling families about SBDC was usually considered a shared activity among multiple providers, including the clinician (*n* = 109, 93%) and DCES (*n* = 104, 90%), followed by the social worker (*n* = 59, 51%). Individuals at small practices were more likely to rely on one provider type (*n* = 14, 40%) compared to medium (*n* = 6, 15%) or large (*n* = 13, 18%) practices (*p* = 0.014). Individuals at small practices were also less likely to rely on a DCES (69% of small practices vs 79% of medium and 90% of large practices, *p* = 0.019) or social worker (20% of small practices vs 43% of medium and 51% of large practices, *p* = 0.008) for this counseling.

Over half of the providers reported discussing SBDC with their patients at multiple visits per year (*n* = 94, 63%). Furthermore, two-thirds of providers reported addressing aspects of SBDC with families between visits by phone or email (*n* = 100, 67%). Clinicians from small and medium practices were more likely to address SBDC with families between visits (*n* = 27, 77% and *n* = 33, 79%, respectively) compared with individuals in large practices (*n* = 40, 56%, *p*=0.02). There were no significant variations by practice environment.

Providers reported positive attitudes towards talking to families about SBDC (Table 2). Most agreed that they are comfortable providing counseling (*n* = 135, 91%), know what questions to ask families (*n* = 115, 78%), can troubleshoot issues with SBDC (*n* = 107, 72%), and have readily available family resources (*n* = 105, 70%). Fewer agreed they had sufficient time (*n* = 73, 49%) or were knowledgeable about federal/state regulations pertaining to T1D management in school (*n* = 69, 46%). Similarly, providers indicated a high degree of comfort counseling families about specific SBDC topics, though they were less comfortable with school regulations, such as 504 Plans and Individualized Education Plans (Table 3). In subanalyses, respondents from large practices and academic environments tended to have lower scores for these questions, indicating less comfort with counseling on some SBDC topics.

### 3.3. Communicating with School Staff for SBDC

Respondents agreed that it was very or extremely important to communicate with school personnel for their patients (*n* = 132, 89%), though the degree of communication varied. Approximately half reported speaking to the school a few times a year (*n* = 84, 56%); 24% reported never communicating with school staff in written or verbal forms.

Similar to counseling families, 79% (*n* = 117) of respondents reported that school communication was typically a shared activity by multiple providers. Most commonly, school communication was the responsibility of DCES (*n* = 108, 92%) or clinician (*n* = 91, 78%) and less commonly of social workers (*n* = 53, 45%) or staff nurses (*n* = 49, 42%). If only one person communicated with school personnel, it was most often a DCES (*n* = 20, 63%). Also, like family counseling, individuals working in small practices more often reported that they had only one person available to communicate with schools (*n* = 13, 37%) than medium (*n* = 6, 14%) or large practices (*n* = 13, 18%) (*p*=0.03) though there was no difference by environment.

Most school staff communication was centered around clarifying school orders (*n* = 118, 79%), answering urgent medical questions (*n* = 113, 76%), or discussing general concerns about the child's T1D management (*n* = 110, 74%). Fewer reported obtaining school blood glucose records (*n* = 49, 33%) or feedback about the student's school T1D management (*n* = 46, 31%). Additionally, a small proportion reported having a school or private duty nurse ever attend patients' medical appointments (*n* = 20, 13%).

Approximately two-thirds of providers reported that their practice offered T1D training to school nurses (*n* = 95, 64%). Though many did offer training, close to one-third (29%) thought that T1D centers should not be responsible for training school nurses. Alternative sources for school nurse education on T1D were felt to be the school district (*n* = 84, 58%), school nursing organizations (*n* = 80, 55%), or diabetes organizations (*n* = 76, 52%), such as the ADA or JDRF.

### 3.4. Barriers & Facilitators to Supporting SBDC

The most significant barriers to supporting SBDC pertained to insufficient resources (Table 4), including available time (82%) and staff to communicate with schools (67%). Other frequently reported barriers related to the school environment, including school nurse education needs (62%) and differences in school policies or practices across districts (70%). Even though providers were less comfortable with the legal statutes pertaining to SBDC, they generally did not consider this to be a significant barrier (28%), except among individuals working in large practices or academic environments (*p* < 0.05 for all).

The surveyed providers overall felt many potential factors would be helpful to facilitate improved SBDC: accessible training for school staff (95%), electronic forms which could be transmitted to the school (90%), regular feedback about school T1D management (89%), feedback about blood glucoses and insulin dosing during school (86%), and a dedicated school liaison (84%). A little over half thought school nurse attendance at visits (60%) may be helpful and desired clinician-focused education about SBDC (57%).

## 4. Discussion

In this national survey, pediatric diabetes care providers, including physicians and APPs, universally agreed that SBDC is very important for their patients and is often a focus both during regular diabetes appointments and between visits. Furthermore, providers universally agreed that communicating with school health staff is highly important. There was variability in how many diabetes care team members are involved in SBDC, the types of team members involved in SBDC, and the frequency that SBDC is addressed both with families and school health staff. The practice size and environment, whether academic or nonacademic, both influenced provider experiences with SBDC counseling and communication with schools. Our findings have important implications for future intervention work targeting school-provider communication and clinical applications to improve SBDC for children with type 1 diabetes.

Providers reported commonly addressing SBDC with patients and school health staff throughout the school year, often carried out by multiple members of the diabetes care team. This is in some contrast with prior studies with parents and school nurses, who have reported suboptimal communication between schools and health-care providers [12–17]. A recent assessment using data from the National Survey of Children's Health reported on parent-perceived communication between their child's clinician and school health team for children with complex medical needs. Though limited communication was even more commonly reported for children with other chronic health conditions, one third of parents of children with T1D reported no communication between their child's school health team and diabetes provider during the school year [17]. This highlights a possible discrepancy between school, parent, and provider perceptions on the frequency and quality of communication for coordination of care.

Providers universally agreed that communicating with school health staff is highly important. Though the respondents perceived frequent communication with school health staff, they also reported that they are not routinely seeking input from school personnel. Obtaining school glucose and insulin dosing records may be less important with the increasing utilization of diabetes devices [26]. However, school nurses have other valuable input. They observe students' self-management abilities and their glucose variability with meals and activities; as a result, they feel they have important contributions to diabetes care [16]. Though privacy concerns may interfere with communication, few respondents identified obtaining parental consent to talk to school staff to be a challenge. It may be that other diabetes care team members are obtaining this feedback, though the survey responses do not suggest that this information reaches the clinician seeing the patient routinely, as close to 90% of respondents wanted more input from school nurses. A formal approach to obtaining school input may facilitate collaboration.

Prior work has identified school- and parent-related barriers to optimal communication among caregivers, including school nurse availability, school district resources, and parent engagement [27, 28]. Our findings offer other insights into what providers perceive to be systems-level factors which impact efforts to improve coordination of SBDC. In addition to staffing and time pressures, internal practice characteristics influenced comfort with counseling. Providers reported significant variability in how their center or practice managed SBDC communications. Respondents working in large practices or academic environments tended to have lower comfort in counseling families about SBDC, and they more often identified their personal knowledge about SBDC as a barrier. This likely relates to practice expectations. Respondents working in these settings also tended to report a multidisciplinary approach to counseling and communicating with schools, indicating that they rely on other team members (DCES, social workers) to bridge gaps in their interactions with patients on these topics.

Additionally, external factors influenced providers' current practices and attitudes. Respondents scored lower in their familiarity with federal and state laws. Knowledge of these laws is important to safeguard the rights of children with T1D in school. Parents and teens have identified concerns about sufficiently trained school staff, permissions to self-manage in school, and school health oversight for field trips [15, 29–31], and legal battles continue to arise, as demonstrated by a recent court decision in New York [32]. Providers need to be prepared to have discussions with families facing possible discriminatory practices in school and direct them to appropriate resources. Furthermore, state law determines school nurse staffing and regulatory supports for T1D, and individual school districts may impose additional policies. These factors may be difficult for busy clinical practices to navigate, which is another limiting factor in offering uniform solutions to implement changes for SBDC.

Taken together, strategies to improve school-provider communication and SBDC should be tailored to the practice and local regulatory landscape. We would consider several possibilities in conjunction with current efforts. Some practices may dedicate a part-time school liaison DCES to establish general guidance for SBDC, train school nurses, and consult on student-specific challenges, like the state-funded diabetes resource nurses in Colorado [33]. Using a telementoring model may be helpful to reach school nurses in rural or under-resourced areas [34]. Depending on the state, one-on-one SBDC nurse education visits would generate billable encounters to formalize in-depth care plans with parents and school nurses, hopefully reducing urgent phone calls between schools and diabetes teams. Lastly, better technology to securely streamline communication between schools and health systems with parental consent may reduce the time burden associated with documentation and faxing records. For future research, the design of SBDC interventions should account for the perspectives of different members of the diabetes care team, in addition to school nurses and parents.

Our study is strengthened by the inclusion of a diverse sample representing different practice environments across the United States. However, with the wide distribution through three organizations to maximize our responses, we cannot calculate a response rate. Additionally, our findings may reflect the opinions of providers who are more invested in SBDC, as they may have been more likely to respond. This may in part explain the high proportion who engage in school-based communication personally and influence the recognized importance of the queried barriers and facilitators to participating in SBDC. Though our open-ended response options did not capture additional barriers or strategies to engage providers and schools, further exploration in a qualitative study should be considered. Importantly, our findings highlight the shared role of DCES or other team members working with clinicians in SBDC management in larger practices. We attempted to survey DCES but received too few responses (*n* = 11) for meaningful analysis. Lastly, we do not know how many of the surveyed providers may come from the same practice or institution, which may influence our findings; however, we intentionally sought individual perspectives. A systems-level review of practices, barriers, and facilitators to school-provider coordination, incorporating the perspectives of DCES and other key stakeholders, will be pursued in future work.

In summary, though providers universally agree that counseling families about SBDC and communicating with schools are important for children with T1D, there are nationwide practice variations, likely influenced by the complexities of local regulations and available resources at individual sites. Interventions to improve SBDC through enhanced school-provider partnerships will require an appreciation of the unique capabilities of not only school systems but also diabetes centers or practices, in different environments. Interventions will need to be tailored or adapted based upon the local context to have far-reaching impact for the health and well-being of children with T1D.

## Figures and Tables

**Table 1:** Self-reported descriptions of respondents' diabetes practices or centers.

Characteristics	*N* (%)
Region (*n* = 144)	
Northeast	14 (10)
Mid-Atlantic	19 (13)
Midwest	41 (29)
Plains	19 (13)
Southeast	29 (20)
Pacific west	22 (15)
Environment (*n* = 148)	
Academic	117 (79)
Nonacademic	31 (21)
Size of practice	
Small <500 patients	35 (24)
Medium, 500–999 patients	42 (28)
Large, >1000 patients	72 (48%)
Types of staff at practice^*∗*^	
Diabetes physician	146 (98)
Advanced practice provider	107 (72)
Diabetes care and education specialist	132 (89)
Dietician	135 (91)
Social worker	106 (71)
Psychologist	82 (55)
Research coordinator	72 (48)
Care manager	26 (17)
Years of experience (*n* = 133)	
<1 year	10 (8)
1–5 years	29 (22)
6–10 years	18 (14)
11–20 years	26 (20)
>20 years	32 (24)
In training	18 (14)

Notes: Characteristics with less than 100% response (*n* = 149) are indicated in the table. Regions were determined as follows: Northeast contains Connecticut, Maine, New Hampshire, Rhode Island, and Vermont. Mid-Atlantic contains Delaware, the district of Columbia, Maryland, New Jersey, New York, Pennsylvania, Virginia, and West Virginia. Midwest contains Illinois, Indiana, Iowa, Kentucky, Ohio, Michigan, Minnesota, Missouri, and Wisconsin. Plains contain Colorado, Kansas, Montana, Nebraska, New Mexico, North Dakota, Oklahoma, South Dakota, and Texas. Southeast contains Alabama, Arkansas, Florida, Georgia, Louisiana, North Carolina, South Carolina, Tennessee, and Puerto Rico. Pacific west contains Alaska, Arizona, California, Hawaii, Idaho, Nevada, Oregon, Utah, and Washington. ^*∗*^Respondents may check all that apply.

**Table 2:** Provider attitudes towards counseling families for school-based diabetes care.

Statement	Overall	Practice size			*p* value	Practice environment		*p* value
		Small	Medium	Large		Academic	Nonacademic	
I am comfortable counseling patients/families about school management of type 1 diabetes	4.29 ± 0.80	4.34 ± 0.91	4.57 ± 0.63	4.10 ± 0.80	**0.001**	4.24 ± 0.76	4.45 ± 0.93	**0.03**
I have the time to address school-based diabetes care during diabetes clinic visits	3.32 ± 1.09	3.46 ± 1.27	3.33 ± 1.16	3.25 ± 0.95	0.50	3.20 ± 1.06	3.81 ± 1.08	**0.005**
I know what questions to ask families about school diabetes care (*n* = 148)	3.94 ± 0.84	4.09 ± 0.85	4.07 ± 0.75	3.79 ± 0.88	0.09	3.86 ± 0.83	4.23 ± 0.84	**0.02**
I can help families troubleshoot issues with school-based diabetes care (*n* = 148)	3.84 ± 0.85	3.94 ± 0.91	4.07 ± 0.75	3.67 ± 0.86	**0.04**	3.75 ± 0.86	4.20 ± 0.75	**0.008**
I have sufficient knowledge about state and federal regulations about diabetes care in school	3.19 ± 1.12	3.37 ± 1.19	3.57 ± 0.94	2.88 ± 1.11	**0.003**	3.03 ± 1.14	3.74 ± 0.86	**0.001**
I have resources I can provide to my patients and families about diabetes care in school	3.88 ± 0.93	3.74 ± 0.95	3.95 ± 0.99	3.90 ± 0.89	0.54	3.83 ± 0.96	4.03 ± 0.80	0.41

Notes: Overall score for respondents is provided, as well as differences by practice size and environment. Small practices have <500 patients; medium practices 500–999 patients, and large practices ≥1000 patients. Response options were on a Likert scale from 1 = strongly disagree to 5 = strongly agree. Data are presented as mean ± SD. Questions with less than 100% response (*n* = 149) are indicated in the table. Significance was determined by Mann–Whitney *U* test (environment) or Kruskal–Wallis test (size) with a *p* value of <0.05.

**Table 3:** Provider comfort with counseling families about specific school-based diabetes care topics.

Statement	Overall	Practice size			*p* value	Practice environment		*p* value
		Small	Medium	Large		Academic	Nonacademic	
Establishing the American Diabetes Association Diabetes Medical Management plan or equivalent school orders	4.36 ± 0.89	4.43 ± 0.88	4.67 ± 0.65	4.15 ± 0.96	**0.005**	4.30 ± 0.94	4.58 ± 0.62	0.19
Discussing food choices in school	4.14 ± 0.79	4.20 ± 0.80	4.17 ± 0.85	4.10 ± 0.75	0.64	4.07 ± 0.82	4.42 ± 0.62	**0.03**
Frequency of blood glucose checks in school	4.72 ± 0.48	4.80 ± 0.41	4.86 ± 0.35	4.61 ± 0.52	**0.02**	4.67 ± 0.49	4.97 ± 0.18	**0.001**
Identifying and treating urgent problems in school (e.g., hypoglycemia, ketones) (*n* = 147)	4.76 ± 0.43	4.83 ± 0.38	4.80 ± 0.40	4.70 ± 0.46	0.28	4.70 ± 0.46	5.00 ± 0.00	**0.001**
Managing glucoses during physical activity (*n* = 148)	4.60 ± 0.53	4.71 ± 0.46	4.68 ± 0.52	4.50 ± 0.56	0.07	4.53 ± 0.55	4.90 ± 0.30	**0.003**
Recommendations for parents remotely monitoring their child's continuous glucose monitor in school	4.34 ± 0.75	4.54 ± 0.56	4.38 ± 0.85	4.22 ± 0.75	0.09	4.26 ± 0.78	4.61 ± 0.56	**0.002**
Recommendations for school nurse use of diabetes devices (e.g., insulin pump, continuous glucose monitor)	4.32 ± 0.79	4.41 ± 0.71	4.40 ± 0.85	4.22 ± 0.79	0.23	4.25 ± 0.82	4.54 ± 0.61	**0.02**
When to consider independent diabetes management by student (*n* = 148)	4.35 ± 0.75	4.40 ± 0.77	4.54 ± 0.55	4.21 ± 0.81	0.13	4.22 ± 0.77	4.84 ± 0.37	**<0.001**
Student's confidentiality and right to privacy in school	3.98 ± 1.02	4.11 ± 0.99	4.10 ± 0.91	3.85 ± 1.08	0.38	3.91 ± 1.02	4.23 ± 0.99	0.09
Establishing a 504 plan or individualized education plan (IEP) if needed (*n* = 148)	3.72 ± 1.11	3.77 ± 1.09	4.15 ± 1.01	3.46 ± 1.10	**0.003**	3.66 ± 1.15	3.90 ± 0.91	0.42

Notes: Overall score for respondents is provided, as well as differences by practice size and environment. Small practices have <500 patients, medium practices 500–999 patients, and large practices ≥1000 patients. Response options were on a Likert scale from 1 = extremely uncomfortable to 5 = extremely comfortable. Data are presented as mean ± SD. Questions with less than 100% response (*n* = 149) are indicated in the table. Significance was determined by Mann–Whitney *U* test (environment) or Kruskal–Wallis test (size) with a *p* value of <0.05.

**Table 4:** Provider perceived barriers to supporting school-based diabetes care (including counseling families and communicating with schools).

Statement	Overall	Practice size			*p* value	Practice environment		*p* value
		Small	Medium	Large		Academic	Nonacademic	
Available resources, including staff, at my center/practice to communicate with schools (*n* = 147)	2.98 ± 1.24	3.03 ± 1.36	3.25 ± 1.28	2.80 ± 1.15	0.18	3.05 ± 1.19	2.74 ± 1.41	0.26
Available time (*n* = 147)	3.57 ± 1.09	3.66 ± 1.16	3.63 ± 0.98	3.50 ± 1.13	0.76	3.61 ± 1.07	3.45 ± 1.18	0.54
My knowledge about general recommendations for school-based diabetes care (*n* = 147)	1.64 ± 0.91	1.40 ± 0.81	1.55 ± 0.90	1.80 ± 0.94	**0.03**	1.73 ± 0.96	1.32 ± 0.65	**0.02**
My knowledge of state and federal regulations about diabetes care in school (*n* = 147)	2.10 ± 1.01	1.91 ± 0.95	1.88 ± 0.91	2.33 ± 1.06	**0.03**	2.23 ± 1.05	1.71 ± 0.74	**0.02**
My knowledge of educational resources for school personnel (*n* = 148)	2.35 ± 1.00	2.20 ± 1.05	2.22 ± 0.96	2.50 ± 0.98	0.16	2.45 ± 0.99	2.00 ± 0.97	**0.02**
Obtaining written parent consent to talk to school personnel (*n* = 148)	1.71 ± 0.96	1.86 ± 1.06	1.68 ± 0.96	1.65 ± 0.91	0.61	1.72 ± 0.99	1.68 ± 0.83	0.84
Parent preferences regarding diabetes management in school that are outside the norm of practice guidelines (*n* = 147)	2.40 ± 1.02	2.14 ± 0.94	2.49 ± 1.00	2.48 ± 1.07	0.28	2.43 ± 1.04	2.32 ± 1.01	0.60
Ease of communicating with school personnel (*n* = 148)	2.63 ± 1.10	2.60 ± 1.21	2.51 ± 1.00	2.71 ± 1.09	0.62	2.72 ± 1.05	2.26 ± 1.18	**0.02**
School nurse training/education about diabetes (*n* = 146)	2.86 ± 1.00	2.91 ± 1.14	2.80 ± 0.98	2.86 ± 0.95	0.79	2.86 ± 0.94	2.84 ± 1.21	0.71
Differences in policies, practices, or resources across schools/school districts (*n* = 125)^*∗*^	2.99 ± 1.01	2.96 ± 1.02	2.97 ± 1.15	3.02 ± 0.94	0.90	3.05 ± 0.96	2.81 ± 1.20	0.33

Notes: Overall score for respondents is provided, as well as differences by practice size and environment. Small practices have <500 patients, medium practices 500–999 patients, and large practices ≥1000 patients. Response options were on a Likert scale from 1 = not at all a significant barrier to 5 = extremely significant barrier. Data are presented as mean ± SD. Questions with less than 100% response (*n* = 149) are indicated in the table. Significance was determined by Mann–Whitney *U* test (environment) or Kruskal–Wallis test (size) with a *p* value of <0.05. ^*∗*^Question was added at the request of one participating organization partway through distribution; thus, only a partial response rate is available.

## Data Availability

The survey data used to support the findings of this study are available from the corresponding author upon request.

## Supplementary Materials

Supplementary Materials A copy of the survey used for this research study is included in the online supplement.
