# Supplementary material for: School-Based Diabetes Care: A National Survey of U.S. Pediatric Diabetes Providers
Source: Pediatr Diabetes. 2023 Apr 11;2023:4313875. doi: 10.1155/2023/4313875 (PMC10624000; doi:10.1155/2023/4313875)
Supplement: Supplementary Materials — A copy of the survey used for this research study is included in the online supplement. [file 4313875.f1.zip › COI Form_March.docx]

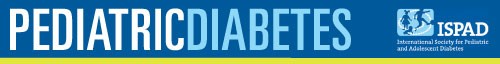


**CONFLICT OF INTEREST DISCLOSURE**

The Editors of *Pediatric Diabetes* recognize that most studies cost money and thus pose a potential

conflict of interest. As a conflict of interest may affect the assessment or judgment of an author, we ask that

**all** authors (not just the Corresponding Author) complete the following form.

For Co-authors: Please complete questions 4-10. Completed forms should be saved, and emailed as an attachment to the Corresponding Author.

For Corresponding Authors: Please complete all questions. It is the responsibility of the Corresponding Author to submit completed forms on behalf of all co-authors via Manuscript Central at the point of manuscript submission.

# Corresponding author only (Co-authors go to Question 4):

POTENTIAL STUDY INTERPRETATION CONFLICTS

1. Some or all of the data that were used in this study were provided by a company with a vested interest in the product being studied. No
2. The sponsor of this project had the right of commenting but the authors retained the right to accept or reject comments or suggestions. n/a
3. The sponsor of this project had the right of final editing and/or approval of the manuscript submitted. n/a

# Corresponding author and Co-authors:

POTENTIAL FINANCIAL CONFLICTS

1. I, my spouse, or one of my dependent children is an employee of a company whose product is being studied. No
2. I, my spouse, or one of my dependent children has significant equity interest (>USD 10,000) in the company that owns the product being studied. No
3. In the past three years I have:

- been paid as a consultant (or in a similar capacity) by a company with a vested interest in the product being studied, on issues related to the product being studied; No
- been paid as a consultant (or in a similar capacity by a company with a vested interest in the product being studies, on issues unrelated to the product being studied; No
- received research or educational support from a company with a vested interest in the product(s) being studied. No

1. A company whose product is being studied has provided funding to support the work on this project. No

If you have answered YES to any of the above questions, or if you have additional personal, commercial or academic conflicts of interest, please draft a statement to publish with the article. e.g., AB has been reimbursed by Safe Drug Ltd. for international conference attendance.

1. Manuscript title:

School-based diabetes care: A national survey of U.S. pediatric diabetes providers

1. Author’s full name (a separate form must be submitted for each author)

Christine March

1. In checking this box, I confirm I have completed this form to the best of my knowledge.

This form is available online by [clicking here](https://onlinelibrary.wiley.com/page/journal/13995448/homepage/forauthors.html)
